# Hippocampus Functional Connectivity, Impulsivity, and Subsequent Substance Use

**DOI:** 10.31586/jcn.2025.1250

**Published:** 2025-03-09

**Authors:** Shervin Assari, Alexandra Donovan, Babak Najand, Golnoush Akhlaghipour, Mario F Mendez

**Affiliations:** 1 Department of Internal Medicine, Charles R. Drew University of Medicine and Science, Los Angeles, CA, USA; 2 Department of Family Medicine, Charles R. Drew University of Medicine and Science, Los Angeles, CA, USA; 3 Department of Urban Public Health, Charles R. Drew University of Medicine and Science, Los Angeles, CA, USA; 4 Marginalization-Related Diminished Returns (MDRs) Center, Los Angeles, CA, USA; 5 Department of Neurology, University of California Los Angeles (UCLA), Los Angeles, CA, USA; 6 Department of Psychiatry & Biobehavioral Sciences, University of California Los Angeles (UCLA), Los Angeles, CA, USA

**Keywords:** Hippocampus, Resting-State Functional Connectivity, Impulsivity, Substance Use, ABCD Study, Adolescence, Tobacco, Marijuana

## Abstract

**Background:**

The hippocampus plays a critical role in memory and decision-making processes, with its resting-state functional connectivity (rsFC) linked to various behavioral outcomes. This study investigates whether baseline brain-wide rsFC of the hippocampus mediates the relationship between impulsivity and subsequent substance use, specifically tobacco and marijuana use, in adolescents.

**Methods:**

Data were drawn from the baseline wave of the Adolescent Brain Cognitive Development (ABCD) study. Resting-state fMRI data were used to evaluate the functional connectivity of the hippocampus with key brain networks, including the cingulo-parietal network, visual network, sensory-motor network, and default mode network (DMN). Impulsivity was assessed using validated self-report measures, and substance use (tobacco and marijuana) was evaluated at follow-up. Mediation models were conducted to examine the extent to which hippocampal rsFC explains the association between impulsivity and substance use.

**Results:**

Baseline hippocampal rsFC with the cingulo-parietal network, visual network, sensory-motor network, and DMN showed marginal associations with future tobacco and marijuana use. Additionally, hippocampal rsFC was significantly associated with impulsivity, which, in turn, predicted higher substance use at follow-up. These findings suggest that hippocampal rsFC partially mediates the relationship between impulsivity and substance use behaviors.

**Conclusions:**

Hippocampal functional connectivity with brain networks may influence the pathway from impulsivity to future substance use in adolescence. These findings emphasize the importance of hippocampal connectivity in understanding the neural mechanisms underlying risk behaviors and may inform the development of targeted interventions to reduce substance use in this vulnerable population.

## 1. Introduction

The hippocampus plays a critical role in memory processing, serving as a key structure for the registration, storage, and retrieval of information [1]. Located in the medial temporal lobe, the hippocampus works in close coordination with surrounding regions such as the parahippocampal area, neocortical association areas, amygdala, hypothalamus, and mammillary bodies. These connections allow the hippocampus to integrate sensory inputs—olfactory, visual, auditory, and tactile—into cohesive memory representations [1–3]. Through its high connectivity, particularly via the fornix to the anterior thalamus and hypothalamus, the hippocampus contributes to memory encoding, consolidation, and retrieval [4]. Its hyperexcitable nature facilitates sustained activity, enabling the transition of short-term memory into long-term memory [5–7]. Beyond its role in human cognition, the hippocampus also plays a survival function in lower animals, guiding decisions related to food, safety, reproduction, and other critical behaviors through olfactory discernment and memory consolidation. These diverse functions underscore the hippocampus’s integral role in both complex decision-making and fundamental survival mechanisms [4].

Research has suggested that the interaction between long-term memory systems and brain reward have implications for adolescents risk behaviors [8]. As shown by, Davidow and colleagues [9], adolescents show a stronger link between reinforcement learning and episodic memory for reward than adults. This behavioral effect is shown to be associated with stronger functional connectivity (FC) between the striatum and the hippocampus during reward learning, which may reflect enhanced reward modulation of long-term memory during adolescence^9,10^. This is because in addition to the VS and other component of the reward system, the hippocampus is required for value-based learning [10]. The VS, hippocampus, and VTA are believed to form a critical reward-memory loop that signals the motivational significance of events and modulates episodic memory formation in the hippocampus [11,12]. The VS-hippocampus FC predicts individual differences in reward modulation of memory [13]. VS-hippocampal circuitry has also been implicated in context-dependent decision making [14] and the formation and retrieval of drug-environment associations, in particular [15,16]. Therefore, individual differences in FC between VS, hippocampus may relate to a propensity for further exploration of substance use among adolescents.

### 1.1. Aim and Hypothesis

This study aims to investigate whether resting-state functional connectivity (rsFC) of the hippocampus is associated with impulsivity and subsequent substance use in adolescents. Specifically, we hypothesize that baseline rsFC between the hippocampus and key brain networks—including the cingulo-parietal network, visual network, sensory-motor network, and default mode network (DMN)—predicts future tobacco and marijuana use. Additionally, we propose that the hippocampal rsFC partially explains the pathway through which impulsivity influences substance use behaviors. By addressing this hypothesis, the study seeks to elucidate the neural mechanisms underlying risk behaviors in adolescence, providing a foundation for developing targeted interventions to mitigate substance use in this vulnerable age group.

## 2. Methods

### 2.1. Study Design and Participants

This study utilized data from the Adolescent Brain Cognitive Development (ABCD) Study, a longitudinal, multisite cohort study designed to explore factors influencing brain development and health in youth across the United States. We analyzed baseline data collected from a diverse sample of children aged 9–10 years, focusing on demographic, social, and neuroimaging variables. The study population included children from a range of racial/ethnic backgrounds and socioeconomic contexts.

### 2.2. Measures

#### Demographic Variables:

Key demographic variables included age (in years) and sex (male vs. female).

### 2.3. Urgency

Positive and negative urgency were measured using the Urgency, Premeditation, Perseverance, Sensation Seeking, Positive Urgency, Impulsive Behavior Scale for Children (UPPS-S [17]. Positive and negative urgency are correlated constructs that reflect different aspects of impulsivity. In this study, positive and negative urgency were treated as continuous measures, with higher scores indicating higher impulsivity and urgency traits. The UPPS-SS is a valid and reliable measure [18].

#### Resting-State Functional Connectivity (rsFC):

Resting-state fMRI data were used to evaluate the functional connectivity of the hippocampus with key brain networks, including the cingulo-parietal network, visual network, sensory-motor network, and default mode network (DMN). These measured were drawn from ABCD data set. ABCD team have calculated the resting state functional connectivity using functional magnetic resonance imaging (fMRI). Standard preprocessing pipelines, including motion correction, spatial normalization, and filtering, were applied to derive connectivity metrics. CON-DMN rsFC was quantified as the Fisher’s z-transformed correlation between mean time series of the two networks.

### 2.4. Harmonization

The MRI procedures used in the ABCD Study are thoroughly explained in detail in other publications [19–24]. The ABCD Imaging Acquisition Workgroup (https://abcdstudy.org/scientists-workgroups.html) developed, refined, and standardized the imaging measures and protocols across all 21 ABCD sites. This referenced work outlines the foundation and methodology of the ABCD imaging protocols and provides an initial assessment of their quality, demonstrating their suitability for children aged 9 to 10 years [25].

### 2.5. Statistical Analysis

Structural equation modeling (SEM) was employed to examine the relationships between demographic variables, rsFC, urgency, and substance use. rsFC, urgency, and substance use were defined as latent factors. Model coefficients (B), standard errors (SE), and p-values were reported for all paths. Age and sex were included as covariates in all models. The SEM allowed both direct effects and indirect effects from age and sex to rsFC and from impulsivity to substance use. Model fit was assessed using the Comparative Fit Index (CFI), Tucker-Lewis Index (TLI), and Root Mean Square Error of Approximation (RMSEA) [26]. A CFI and TLI above 0.90 and an RMSEA below 0.08 indicated acceptable model fit. Missing data were handled using full information maximum likelihood estimation. All analyses were conducted using Stata 18.0.

### 2.6. Ethical Considerations

The ABCD Study protocol was approved by the institutional review board of the UCSD. Informed consent and assent were obtained from parents and children, respectively. Data were analyzed anonymously.

## 3. Results

### 3.1. Sample Characteristics

Table 1 presents the summary of the data. The study analyzed data from 11,733 participants aged 9.48 years on average (SE=0.006SE = 0.006). The sample included slightly more males (52.14%, n=6,181) than females (47.86%, n=5,673). Key descriptive statistics are summarized below.

#### Resting-State Functional Connectivity (rsFC):

Mean rsFC values with the cingulo-parietal network (M=0.154,SE=0.002), default mode network (M=0.072,SE=0.002), sensory-motor network (M=0.076,SE=0.002), and visual network (M=0.055,SE=0.002) reflect hippocampal connectivity across these regions.

#### Impulsivity Measures:

Positive urgency had a mean score of 8.009 (SE=0.033), while negative urgency had a mean score of 8.508 (SE=0.029).

#### Substance Use Prevalence:

- **Marijuana Use:** The majority of participants (96.59%, n=11,333) reported no marijuana use, while 3.41% (n=400) indicated use.
- **Tobacco Use:** Most participants (94.73%, n=11,115) reported no tobacco use, while 5.27% (n=618) had used tobacco.

### 3.2. Bivariate Correlations

The bivariate correlations between study variables, including demographic factors (age and sex), hippocampal resting-state functional connectivity (rsFC) with specific networks, impulsivity (positive and negative urgency), and future substance use (tobacco and marijuana), are summarized in Table 2.

1. Demographic Variables:
  - Age was positively correlated with future tobacco use (r=0.073,p<0.001) and future marijuana use (r=0.075,p<0.001), indicating that older adolescents are more likely to engage in substance use.
  - Sex (male) showed a weak positive correlation with negative urgency (r=0.089,p<0.001) and a weak positive correlation with positive urgency (r=0.076,p<0.001).
2. Hippocampal rsFC:
  - Right cingulo-parietal rsFC was positively correlated with left default mode rsFC (r=0.127,p<0.001) and left sensory-motor rsFC (r=0.138,p<0.001), reflecting coherence between hippocampal connectivity across networks.
  - Left default mode rsFC was positively correlated with left visual rsFC (r=0.084,p<0.001) and left sensory-motor rsFC (r=0.042,p<0.001).
  - Left sensory-motor rsFC showed a significant correlation with left visual rsFC (r=0.075,p<0.001).
3. Impulsivity and Substance Use:
  - Positive urgency (UPPS) was significantly associated with future tobacco use (r=0.039,p<0.001) and future marijuana use (r=0.040,p<0.001).
  - Negative urgency (UPPS) demonstrated a stronger association with future tobacco use (r=0.035,p=0.001) and marijuana use (r=0.028,p=0.008).
  - Positive and negative urgency were strongly correlated (r=0.503,p<0.001), indicating shared variance between these impulsivity traits.
4. Substance Use Correlations:
  - Future tobacco use and future marijuana use were strongly correlated (r=0.446,p<0.001), suggesting overlap in risk factors for these behaviors.

**Figure 1.**
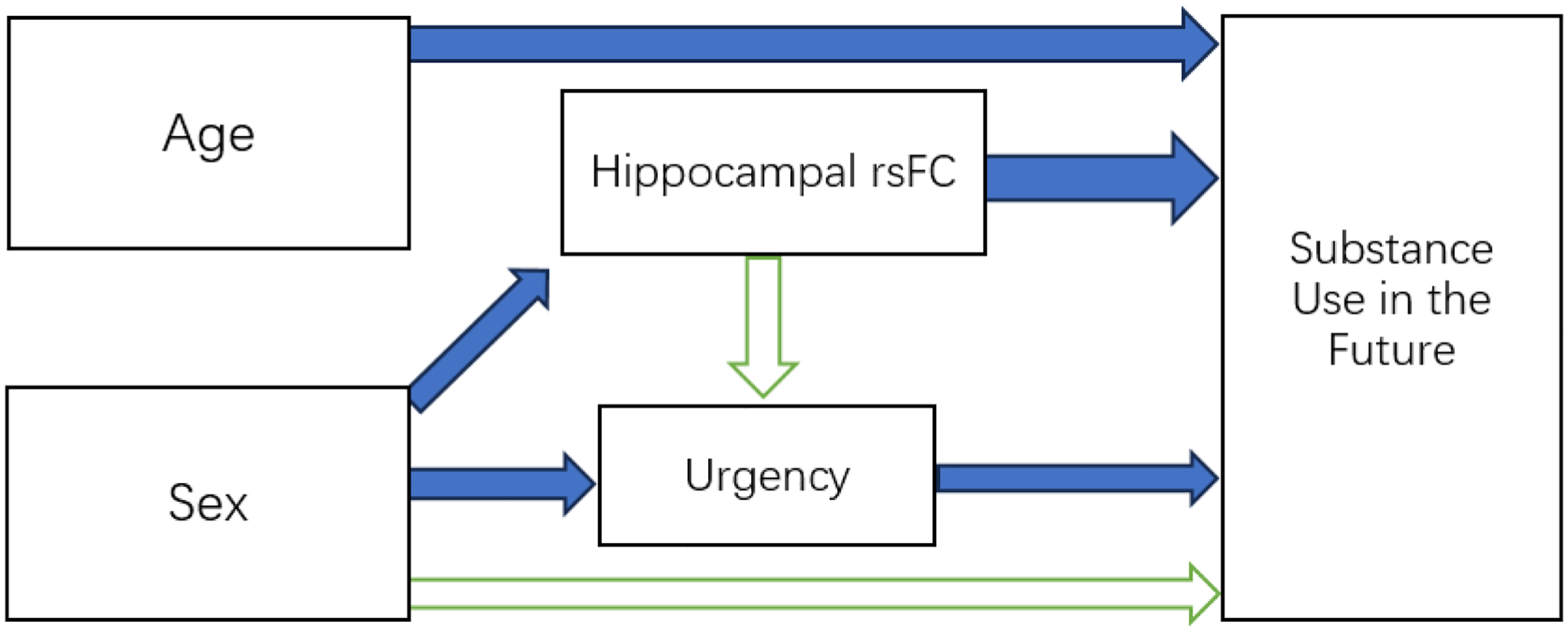
Summary of structural equation modeling (SEM); ***Note:*** *Green Arrows: Non-Significant, Blue Arrows: Statistically or Marginally Significant*

Figure 1 and Table 3 show the results of the structural equation modeling (SEM) analysis examined the relationships among hippocampal resting-state functional connectivity (rsFC), urgency, and future substance use, while accounting for age and sex as covariates. The results indicate that urgency mediates the relationship between age and future substance use, while hippocampal rsFC shows a weak direct association with substance use and urgency. Male sex was a significant predictor of hippocampal rsFC and urgency but not future substance use. These findings highlight the complex interplay between neurobiological, psychological, and demographic factors in predicting substance use behaviors. To be more specific, these paths were found:

#### Hippocampus rsFC

Male sex was significantly associated with hippocampal rsFC (B=0.500, 95% CI [0.484, 0.516], p<0.001), indicating higher connectivity in males.

#### Urgency

Hippocampal rsFC did not significantly predict urgency (B=−0.015, 95% CI [−0.061, 0.031], p=0.524).

Age was positively associated with urgency (B=0.205, 95% CI [0.199, 0.210], p<0.001).

Male sex was also significantly associated with higher urgency (B=0.123, 95% CI [0.097, 0.149], p<0.001).

#### Substance Use in the Future

Hippocampal rsFC showed a marginal association with future substance use (B=−0.039, 95% CI [−0.083, 0.005], p=0.082).

Urgency significantly predicted future substance use (B=0.052, 95% CI [0.019, 0.084], p=0.002).

Age also showed a positive association with future substance use (B=0.008, 95% CI [0.001, 0.016], p=0.018).

Male sex was not significantly associated with future substance use (B=−0.012, 95% CI [−0.037, 0.013], p=0.362).

## 4. Discussion

This study provides important insights into the role of hippocampal resting-state functional connectivity (rsFC) in predicting future substance use, specifically tobacco and marijuana, in adolescents. Using data from the Adolescent Brain Cognitive Development (ABCD) study, we found that baseline hippocampal rsFC with key brain networks, including the cingulo-parietal network, visual network, sensory-motor network, and default mode network (DMN), was associated with future substance use. Additionally, impulsivity traits were associated with higher substance use, supporting the role of neurocognitive and behavioral factors in adolescent risk behaviors. These findings emphasize the importance of hippocampal connectivity in understanding the neural mechanisms underlying substance use during adolescence.

### 4.1. Hippocampal Connectivity and Risk Behaviors

The hippocampus plays a central role in integrating sensory, cognitive, and emotional information, making it essential for decision-making and behavioral regulation. In this study, hippocampal rsFC with the DMN, sensory-motor, and visual networks was associated with future substance use. Connections with the DMN, which is implicated in self-referential thought and future planning, may influence adolescents’ ability to reflect on the long-term consequences of substance use. Similarly, sensory-motor and visual network connections may reflect heightened sensitivity to environmental cues, such as those associated with substances, which can drive risk behaviors. These findings align with prior research linking hippocampal connectivity with impulsivity and cue-reactivity in substance use contexts.

### 4.2. Network-Specific Findings

The observed associations between hippocampal rsFC and key networks highlight the complexity of brain-wide connectivity patterns underlying substance use behaviors. The cingulo-parietal network, involved in cognitive control and executive functioning, may play a role in adolescents’ susceptibility to risk-taking when its connectivity with the hippocampus is altered. Similarly, the sensory-motor and visual networks may contribute to substance use through heightened reactivity to external stimuli, particularly in social and environmental contexts where substances are present. These findings suggest that hippocampal rsFC reflects a broader network-level vulnerability to risk behaviors, which warrants further exploration.

### 4.3. Adolescence as a Critical Period

Adolescence is a period of rapid neurodevelopment and heightened vulnerability to risk behaviors, including substance use. The associations observed in this study underscore the importance of hippocampal rsFC during this developmental stage. Adolescents with altered connectivity patterns may experience challenges in regulating impulsivity, leading to a greater likelihood of engaging in substance use behaviors. These findings emphasize the need to consider neurodevelopmental factors when designing interventions aimed at preventing substance use during adolescence.

### 4.4. Implications for Prevention and Intervention

The results of this study have significant implications for prevention and intervention strategies targeting adolescent substance use. Interventions aimed at modulating hippocampal rsFC, such as mindfulness training, cognitive-behavioral therapy, or neurofeedback, could help strengthen connectivity with networks involved in cognitive control and decision-making. Additionally, reducing exposure to substance-related cues and enhancing adolescents’ ability to manage impulsivity may further mitigate substance use risk. Advances in neuromodulation techniques, such as transcranial magnetic stimulation (TMS) and transcranial direct current stimulation (tDCS), could offer promising avenues for targeting specific connectivity patterns if future research confirms their safety and efficacy.

### 4.5. Limitations

This study has several limitations that should be acknowledged. First, while rsFC was assessed at baseline and substance use was evaluated at follow-up, the cross-sectional nature of connectivity data limits our ability to establish causality. Second, although the sample was large and diverse, the findings may not generalize to other populations or cultural contexts. Third, this study focused on tobacco and marijuana use, and future research should explore whether similar patterns are observed for other substances, such as alcohol or stimulants. Finally, while this study examined hippocampal rsFC with select brain networks, a more comprehensive analysis of connectivity with other regions and networks could provide additional insights.

### 4.6. Future Research

Future research should focus on incorporating longitudinal neuroimaging data to better understand the developmental trajectories of hippocampal rsFC and their relationship to substance use behaviors. Exploring how these trajectories differ across populations with varying demographic and socioeconomic characteristics could provide a more nuanced understanding of substance use risk. Expanding the scope of research to include additional substances and employing advanced techniques, such as machine learning and graph theory analysis, may enhance the predictive power of connectivity models and inform the development of personalized interventions. Additionally, investigating the potential for neuromodulation to alter rsFC patterns and reduce substance use risk represents an important avenue for future work.

## 5. Conclusions

In conclusion, this study highlights the significant role of hippocampal rsFC in predicting future substance use in adolescents. By identifying key connections with networks such as the DMN, cingulo-parietal, sensory-motor, and visual networks, our findings provide a deeper understanding of the neural underpinnings of risk behaviors. These results underscore the importance of targeting hippocampal connectivity in the development of effective interventions to reduce adolescent substance use and promote healthier developmental outcomes.

## Figures and Tables

**Table 1.** Descriptive Data (n = 11,733)

	Mean	SE
Age (Years)	9.477	0.006
rsFC with Cingulo-parietal	0.154	0.002
rsFC with Default	0.072	0.002
rsFC with Sensorymotor	0.076	0.002
rsFC with Visual	0.055	0.002
Positive urgency	8.009	0.033
Negative urgency	8.508	0.029
	n	%
Marijuana Use		
No	11,333	96.59
Yes	400	3.41
Tobacco Use		
No	11,115	94.73
Yes	618	5.27
Sex		
Female	5,673	47.86
Male	6,181	52.14

**Table 2.** Bivariate Correlations

	1	2	3	4	5	6	7	8	9	10
1 Age	1.000									

2 Sex (Male)	0.021	1.000								
	0.024									

3 Cingulo Parietal (Right)	−0.010	−0.013	1.000							
	0.303	0.187								

4 Default Mode (Left)	−0.019	0.046	0.127	1.000						
	0.051	0.000	0.000							

5 Sensorymotor (Left)	0.006	−0.004	0.138	0.042	1.000					
	0.544	0.704	0.000	0.000						

6 Visual (Left)	−0.001	0.008	0.126	0.084	0.075	1.000				
	0.928	0.411	0.000	0.000	0.000					

7 Positive Urgency (UPPS)	−0.028	0.076	−0.023	−0.003	−0.036	−0.031	1.000			
	0.006	0.000	0.036	0.824	0.001	0.005				

8 Negative Urgency (UPPS)	−0.012	0.089	0.001	0.001	−0.002	−0.013	0.503	1.000		
	0.238	0.000	0.956	0.904	0.857	0.224	0.000			

9 Tobacco Use (Future)	0.073	−0.013	−0.024	−0.001	−0.011	−0.003	0.039	0.035	1.000	
	0.000	0.165	0.018	0.930	0.267	0.778	0.000	0.001		

10 Marijuana Use (Future)	0.075	0.005	−0.003	−0.005	−0.008	−0.013	0.040	0.028	0.446	1.000
	0.000	0.605	0.730	0.597	0.440	0.201	0.000	0.008	0.000	

**Table 3.** Summary of SEM

			B	SE	95%		p
Structural							
Predictor		Outcome					
Sex (Male)	→	Hippocampus rsFC	0.500	0.008	0.484	0.516	0.000


Hippocampus rsFC	→	Urgency	−0.015	0.024	−0.061	0.031	0.524
Age	→	Urgency	0.205	0.003	0.199	0.210	0.000
Sex (Male)	→	Urgency	0.123	0.013	0.097	0.149	0.000


Hippocampus rsFC	→	Substance Use in the Future	−0.039	0.022	−0.083	0.005	0.082
Urgency	→	Substance Use in the Future	0.052	0.017	0.019	0.084	0.002
Age	→	Substance Use in the Future	0.008	0.004	0.001	0.016	0.018
Sex (Male)	→	Substance Use in the Future	−0.012	0.013	−0.037	0.013	0.362

Measurement							

Cingulo Parietal Network (Right)	→	Hippocampus rsFC	0.752	0.009	0.735	0.769	0.000
Default Mode Network (Left)	→	Hippocampus rsFC	0.465	0.009	0.448	0.482	0.000
Sensory Motor Network (Left)	→	Hippocampus rsFC	0.439	0.009	0.421	0.456	0.000
Visual Network (Left)	→	Hippocampus rsFC	0.340	0.009	0.322	0.358	0.000

Positive Urgency	→	Urgency	0.670	.006	0.659	0.681	0.000
Negative Urgency	→	Urgency	0.789	0.007	0.776	0.802	0.000

Tobacco Use	→	Substance Use in the Future	0.748	0.019	0.711	0.784	0.000
Marijuana Use	→	Substance Use in the Future	0.595	0.015	0.566	0.624	0.000
→							
